# Sleep quality mediates the effect of medical social support on depression symptoms in patients with HIV/AIDS

**DOI:** 10.1186/s12889-024-18174-w

**Published:** 2024-05-28

**Authors:** Ziqi Chen, Kailian He, Yulu Chen, Xiashuang Zhang, Zeyan Ye, Chaofan Xie, Tingyu Luo, Xiaofeng Fu, Wuxiang Shi, Zhiyong Zhang, Liang Cao, You Li

**Affiliations:** 1 Department of Environmental Health and Occupational Medicine, School of Public Health, Guilin Medical University, Guilin, Guangxi 541199 People’s Republic of China; 2 Guangxi Health Commission Key Laboratory of Entire Lifecycle Health and Care, Guilin Medical University), Guilin, Guangxi 541199 People’s Republic of China; 3 Health and Wellness Section, Yibin Center for Disease Control and Prevention, Yibin, Sichuan 644600 People’s Republic of China; 4 Department of AIDS Control and Prevention, Guilin Center for Disease Control and Prevention, Guilin, Guangxi 541000 People’s Republic of China; 5 Health Management Unit, Faculty of Humanities and Management, Guilin Medical University, Guilin, Guangxi 541199 People’s Republic of China; 6 Department of Experimental Teaching Center, School of Public Health, Guilin Medical University, Guilin, Guangxi 541199 People’s Republic of China

**Keywords:** HIV/AIDS, Sleep quality, Depression symptoms, Mediating effect

## Abstract

**Objectives:**

The purpose of our study is to further understanding of the depression symptoms of HIV/AIDS patients in Guilin, Guangxi via exploring whether there is a mediating effect of sleep quality on medical-social support and depression symptoms and therefore provide a theoretical basis for application of medical-social support to alleviate depression symptoms of HIV/AIDS patients.

**Methods:**

A convenience sampling method was used to select 200 HIV/AIDS patients for the study. Depression symptoms, sleep quality, and medical-social support of the study participants were investigated using The Center for Epidemiological Studies Depression Scale (CES-D), The Pittsburg Sleep Quality Index (PSQI), and The Medical Outcomes Study Social Support Survey (MOS-SSS), respectively. Predictors of depression symptoms were explored by multiple linear regression, and Pearson correlation was used to analyze the relationship between sleep quality, medical-social support, and depression symptoms. Mediating effect analysis was performed by nonparametric Bootstrap test.

**Results:**

In this study, the incidence of depression symptoms was 54.4%. Multiple linear regression analysis showed that leanness (β = 0.161, *P* = 0.008), obesity (β = 0.186, *P* = 0.002), sleep quality score > 7 (β = 0.331, *P* < 0.001), and medical-social support score > 56 (β = -0.247, *P* < 0.001) could influence depression symptoms of HIV and Pearson’s correlation analysis demonstrated that there was a two-way correlation between sleep quality, medical social support and depression symptoms (*P* < 0.05). In addition, Bootstrap tests showed that medical-social support might affect depression symptoms not only directly but also indirectly through the mediating effect of sleep quality with the direct and mediating effects accounting for 77.25% and 22.75% of the total effect, respectively.

**Conclusion:**

The prevalence of depression symptoms is high among HIV/AIDS patients in Guilin City. The depressive symptoms of PLWHs(people living with HIV) are related to their sleep quality and medical-social support, and sleep quality partially mediates the relationship between medical-social support and depression symptoms. Therefore, interventions to improve sleep quality and medical-social support have the potential to allay the depression symptoms of HIV/AIDS patients.

**Supplementary Information:**

The online version contains supplementary material available at 10.1186/s12889-024-18174-w.

## Introduction

According to a survey conducted by UNAIDS in 2020, about 37.6 million people worldwide have been infected with HIV/AIDS by 2020, and about 480,000 to 1 million people may die from AIDS-related illnesses [1]. The Chinese Health Care Commission also showed that in 2021a total of 60,154 AIDS cases were reported nationwide, of which 19,623 died [2]. Suffice it to say that AIDS has become a serious global public health and social problem which imposes an enormous physical and economic burden on both individuals, health care systems and society. HIV patients may suffer from physical diseases as well as several mental problems, among which depression is the most common-seen mental health challenge,with clinical manifestations as low mood, loss of pleasure, reduced motivation and energy, guilt or low self-esteem, problems with sleep or appetite, suicidal attempt and concentration difficulties [3]. Globally, more than 350 million people currently suffer from depression, and its lifetime prevalence in the general population is estimated around 3 to 17%. Studies had shown that depression was three times more prevalence in the HIV-positive population than in the general population, and its lifetime prevalence this population was estimated to be between 22% and 45% [4]. Predicted by WHO, by the end of 2030, depression and HIV/AIDS will be the leading causes of disability around the world [1].A variety of factors may contribute to the development of depressive symptoms in PLWH, such as age, gender, marital status, monthly income, occupational status, opportunistic infections, adverse reactions to medications, the presence of other chronic diseases, social stigma, occupational disability, low social support, and chronic physical discomfort and illness [5]. Antidepressants are an effective way to treat depression, however, PLWH patients are more likely to receive psychotherapy than medication.

It's held that medical-social support may strengthen the belief and will of HIV patients to survive and have a positive impact on their physical health. A multitude of previous studies had shown that PLWHs often had to endure severe illness-related stress, including stigma, discrimination, financial difficulties, side effects of antiretroviral therapy (ART) treatment, unemployment, etc., any of which could lead to depression, anxiety, suicidality [6]. Studies had shown that adult PLWHs with poor social support were 31% more likely to develop depression than those with more social support. Less social support may result in irregular medication which in turn aggravates the infected status and therefore, causes social isolation, and eventual depression [7]. Another study confirmed a significant negative association between medical-social support and poor sleep quality, with older adults with low levels of medical-social support having a 1.40 times higher risk of poor sleep quality compared to those with high levels of social support [8]. Having positive social relationships can promote healthier sleep by encouraging positive emotions, reducing stress, and preventing social isolation [9]. Social isolation can lead to greater stress and damage mental health [10]. Sufficient medical-social support may enable PLWHs to cope with the negative effects of stress more actively [6].

Sleep disturbance is an often-heard complaint in patients with chronic illnesses, including those with HIV infection. It may occur at any stages of HIV infection, but is more common at the advanced stage of HIV. A meta-analysis showed that the global prevalence of PLWH sleep disorders in adults was 58 percent [11]. Better sleep quality in HIV patients is closely related to the indicators of quality of life, such as overall well-being, anxiety, reduced depressive symptoms, and reduced symptom severity, whereas co-occurrence of poor sleep quality and HIV infection complicates clinical management. Poor sleep quality has been reported commonplace in PLWHs. Studies conducted in the United States and China [12, 13] had shown, the symptom most closely correlating with poor sleep quality was depression. Depression leads to hyperfunction of the hypothalamic-pituitary-adrenal axis, increased sympathetic excitability, and elevated circulating levels of norepinephrine and cortisol, resulting in an enhanced state of arousal in the patient, which in turn affects sleep quality [14]. At the same time, sleep problems can precede and precipitate depressive episodes, which in turn contribute to the development of depression. Even after depressive symptoms have resolved, sleep problems remain a residual symptom that needs to be treated [15]. Thus, there is a strong link between sleep and depressive symptoms, and sleep may even play an important role in the etiology and maintenance of depressive symptoms.

In the specific population of HIV/AIDS patients, the relationship between both sleep quality, medical-social support and depression has been studied more [12, 15, 16], while the mediating role of sleep quality between medical-social support and depression has rarely been examined. The present study aimed to fill this research gap and provide evidence for effective prevention and treatment of depressive symptoms in HIV/AIDS patients in the future. Therefore, we analyzed the possible influences on depressive symptoms among HIV patients in Guilin City, focusing on the relationship between sleep quality, medical-social support and depressive symptoms, and proposed the hypothesis that sleep quality has a mediating effect between medical-social support and depressive symptoms.

## Objects and methods

### Study population

Convenience sampling was adopted in cross-sectional study to select 200 patients with HIV/AIDS in Guilin City from January 2022 to July 2022 for the current study. Inclusion criteria were as follows: (1) Age ≥ 18 years. (2) Those who were tested positive for HIV antibody. (3) Full ability to comprehend and complete the questionnaire independently. Exclusion Criteria were the following: (1) serious visual and hearing impairment or insufficient reading and comprehension ability. (2) serious acute or chronic physical illnesses which resulted in functional impairment. (3) Unwillingness to participate in this survey. This study was conducted by the investigators (all of whom were trained in standardized procedures professional staff engaged in the follow-up of HIV/AIDS patients in Guilin City and various counties and districts) who carried out the questionnaire survey on the selected participants through interviews. Recruitment of participants was limited to those who were followed up in the department of AIDS control and prevention and the confidentiality of the subjects was ensured. All subjects signed an informed consent form prior to the survey, and the survey was conducted under the approval of the Ethics Committee of Guilin Medical College(GYLL2021078).

### Methods

#### Estimation of sample size

According to relevant studies [17], the prevalence of depressive symptoms in HIV-infected patients was about 41.0%, with a permissible error of 0.1 and a Zα of 1.96, which was calculated using the formula n=(Zα^2^ × pq)/d^2^, yielding a sample size of 95. Considering a 10% failure rate and the design effect of the survey (m = 1.5), N = n×(1 + 0.1)×1.5 = 154(persons).

### Data collection

We collected demographic, including the following: (a) General demographic characteristics: basic personal information such as gender, age, ethnicity, height, weight, household location, health status, education level, monthly household income, occupation, marital status, etc.; (b) Clinical information: level of CD4 + T-lymphocyte (referred to as CD4 + cells) counts, HIV viral loads, diagnosis time and infection, antiretroviral treatment, etc.; (c) Other information: route of infection, HIV testing of last year, sexual life within 6 months, history of chronic illness, etc. [16, 18]. According to the Guidelines for the Prevention and Control of Overweight and Obesity in Chinese Adults [19], Body Mass Index (BMI) < 18.5 kg/m^2^ indicates lean, BMI between 18.5 and 23.9 kg/m^2^ indicates normal weight, BMI between 24.0 and 27.9 kg/m^2^ indicates overweight, and BMI ≥ 28.0 kg/m^2^ indicates obesity.

Depression was measured using The Center for Epidemiological Studies Depression Scale (CES-D) [20], designed by the National Institute of Mental Health and widely used in general population for screening and assess depressive symptoms, which was primarily used to measure the degree of depression of the recent week in the study population. The scale is set up from four dimensions which are Depressed Mood, Positive Mood, Somatic Symptoms and Activity Latency. The CES-D scale is scored on a 0–3 Likert 4-point scale, with total scores ranging from 0 to 60. There is no specific and definite score for depression. A score of ≥ 16 is generally considered to display a potential tendency for depression and the higher scores may show a higher likelihood of being depressive.

The Pittsburg Sleep Quality Index (PSQI), compiled by Buysse et al. [21] in 1989 and translated into Chinese by Liu Xianchen et al. [22] in 1996, was verified to have a reliability of 0.85 and a validity of 0.83, which is suitable for measuring sleep condition of the latest month in Chinese population. The PSQI scale consists of seven factors: subjective sleep quality, sleep latency, duration of sleep, habitual sleep efficiency, sleep disorders, medication for sleep, and daytime dysfunction. Each factor is scored from 0 to 3, and the total score of the scale ranges from 0 to 21, with a score of “0” indicating no difficulty and a score of “21” indicating great difficulty in all areas. In Chinese population, a total PSQI score of > 7 indicates presence of sleep disorders [22].

The Medical Outcomes Study Social Support Survey (MOS-SSS), developed by Sherbourne & Stewart [23], consists of four dimensions: practical support, message and emotional support, interactive social cooperation, and emotional support. A score of 1 to 5 is recorded depending on the number of occurrence and the degree of severity, with 1 indicating no support at all, 2 indicating scanty support, 3 indicating support available for some time, 4 indicating support available for most of the time, and 5 indicating constant support. The total score ranges from 19 to 95, and the higher the score is, the higher the level of medical social support patients may receive. Some Chinese scholars proposed that 56 points might be used as a cutoff and ≤ 56 points representing low medical social support while > 56 points might indicate high medical social support [2].

### Statistical analysis

Data were entered in double parallel using EpiData 3.1 software and statistically analyzed by SPSS28.0. Summarized as mean ± standard deviation (‾χ ± s) and categorical variables as frequency (percentage). One-way test, Kolmogorov-Smirnov test was used to assess normal continuous variables, and data conforming to normal distribution were analyzed using t-test or chi-square test. Following the one-way analysis, multiple linear regression was used to further investigate the relationship between the variables of interest and depressive symptoms. Pearson correlations were analyzed for the relationship between sleep quality, medical-social support, and depression symptoms, followed by mediated effects analyses via nonparametric Bootstrap using the SPSS28.0 macro program PROCESS4.1 component. The Bootstrap methodology was developed from the 5000 replicate samples to generate 95% bias-corrected confidence intervals, with intervals excluding 0 indicating a significant mediating effect. Finally, mediation effect plots were further demonstrated by graphing with AMOS 24.0 software. *P* < 0.05 was considered statistically significant.

## Results

### Demographic characteristics

A total of 200 questionnaires were distributed in this study and all 200 questionnaires were retrieved. Among them, 129 (64.50%) were made on male and 71 (35.5%) were on female. The age distribution ranged from 21 to 85 years with a mean age of 51.53 ± 13.44 years. The participants were mainly Han (76.00%), followed by Yao (19.50%), 59.00% had a partner in their marital status. In terms of educational attainment, 39.50% were in elementary school and below, 41.50% in junior high school, and 19.00% in high school and above, and by occupation, 74.00% were farmers. The mean score of depression scale in HIV/AIDS patients was 18.33±9.72 and the incidence of depressive symptoms was 54.40%.The results of univariate analysis showed statistically significant differences in depression across health status, BMI, antiretroviral treatment status, sleep quality and medical-social support (all *P* < 0.05) (Table 1).

**Table 1:** Comparison of general conditions of HIV/AIDS patients

Characteristics	N (%)	Sleep quality	Medical-social support	Depression symptoms
Genders				
Male	129 (64.50)	5.705 ± 4.223	54.977 ± 17.512	17.519 ± 9.547
Female	71 (35.50)	6.718 ± 3.750	56.127 ± 15.634	19.789 ± 9.925
T		-1.687	-0.461	-1.586
*P*		0.093	0.645	0.114
Age, (yeas)				
<50	85 (42.50)	5.612 ± 3.964	55.576 ± 17.673	18.776 ± 8.970
≥ 50	115 (57.50)	6.400 ± 4.150	55.243 ± 16.271	17.991 ± 10.264
T		-1.353	0.138	0.564
*P*		0.178	0.890	0.574
Ethnicity				
Han	152 (76.00)	6.237 ± 4.186	55.118 ± 17.687	17.816 ± 10.029
Yao	39 (19.50)	5.462 ± 3.409	55.615 ± 14.100	20.154 ± 8.509
Others	9 (4.50)	5.778 ± 5.094	58.889 ± 13.724	19.000 ± 9.247
F		0.581	0.216	0.920
*P*		0.560	0.806	0.400
Marital status				
With partner	118 (59.00)	5.415 ± 3.440	57.602 ± 16.226	18.000 ± 9.806
No partner	82 (41.00)	7.000 ± 4.725	52.195 ± 17.287	18.793 ± 9.634
T		-2.597	2.256	-0.566
*P*		0.010	0.025	0.572
Education				
Elementary school and below	79 (39.50)	7.241 ± 4.282	52.152 ± 17.425	19.658 ± 10.646
Junior High School	83 (41.50)	5.422 ± 3.646	55.795 ± 16.164	18.241 ± 9.636
High School and above	38 (19.00)	5.026 ± 4.057	61.211 ± 15.755	15.737 ± 7.258
F		5.807	3.863	2.117
*P*		0.004	0.023	0.123
Career				
Farmers	148 (74.00)	5.939 ± 3.767	55.041 ± 16.537	17.905 ± 9.444
Workers/services	25 (12.50)	6.840 ± 5.528	58.240 ± 19.361	19.280 ± 10.722
Others	27 (13.50)	6.037 ± 4.283	54.630 ± 16.397	19.741 ± 10.424
F		0.519	0.415	0.543
*P*		0.596	0.661	0.582
Health Status				
Poor	13 (6.50)	10.385 ± 4.292	50.077 ± 19.902	26.846 ± 10.439
General	71 (35.50)	7.268 ± 4.323	53.352 ± 15.123	20.761 ± 10.687
Well	116 (58.00)	4.845 ± 3.320	57.224 ± 17.348	15.879 ± 8.041
F		18.246	1.872	12.116
*P*		<0.001	0.157	<0.001
Income (CNY)				
<1000	64 (32.00)	6.328 ± 4.346	52.266 ± 17.862	17.422 ± 11.420
1001 ~ 2000	59 (29.50)	5.763 ± 3.612	55.288 ± 16.565	18.356 ± 8.570
2001 ~ 3000	48 (24.00)	5.396 ± 3.902	57.771 ± 14.206	19.229 ± 9.210
>3000	29 (14.50)	7.207 ± 4.562	58.517 ± 18.652	18.759 ± 8.943
F		1.392	1.397	0.339
*P*		0.247	0.245	0.797
Household Registration				
Rural	161 (80.50)	6.019 ± 3.957	54.373 ± 17.107	18.453 ± 10.008
City	39 (19.50)	6.256 ± 4.610	59.564 ± 15.176	17.795 ± 8.520
T		-0.326	-1.736	0.379
*P*		0.745	0.084	0.705
BMI (kg/m^2^)				
Lean	29 (14.50)	5.966 ± 3.986	54.276 ± 16.349	21.690 ± 10.794
Normal weight	128 (64.00)	6.188 ± 4.091	55.063 ± 17.548	17.406 ± 9.367
Overweight	35 (17.50)	5.171 ± 3.258	58.029 ± 14.199	16.571 ± 8.158
Obesity	8 (4.00)	8.375 ± 6.653	53.000 ± 19.413	28.500 ± 10.406
F		1.468	0.395	5.145
*P*		0.224	0.757	0.002
Route of Infection				
Heterosexual transmission	177 (88.50)	6.051 ± 4.048	55.542 ± 16.627	17.989 ± 9.805
Homosexual transmission	18 (9.00)	6.278 ± 4.638	58.667 ± 17.918	21.167 ± 9.044
Non-sexual transmission	5 (2.50)	5.800 ± 3.962	38.000 ± 12.247	20.000 ± 8.746
F		0.036	3.077	0.949
*P*		0.965	0.048	0.389
Antiviral Treatment Status				
Not treated	43 (21.50)	7.744 ± 4.909	55.930 ± 17.856	21.674 ± 10.884
Received treatment	157 (78.50)	5.605 ± 3.710	55.236 ± 16.606	17.408 ± 9.201
T		2.657	0.239	2.587
*P*		0.010	0.811	0.010
Duration of Infection (years)				
<1	42 (21.00)	7.762 ± 5.169	58.619 ± 18.285	21.071 ± 11.788
1 ~ 2	12 (6.00)	5.917 ± 4.641	57.667 ± 13.013	18.500 ± 13.070
3 ~ 5	42 (21.00)	5.881 ± 3.717	54.619 ± 17.518	17.595 ± 8.402
>5	104 (52.00)	5.471 ± 3.492	54.125 ± 16.363	17.490 ± 8.767
F		3.299	0.811	1.464
*P*		0.021	0.489	0.226
Most Recent CD4 + Count(cells/mm3)				
<200	47 (23.50)	6.915 ± 4.496	55.000 ± 16.926	20.532 ± 11.521
200–409	71 (35.50)	5.972 ± 3.633	55.634 ± 15.972	17.859 ± 9.288
410–1590	82 (41.00)	5.659 ± 4.176	55.390 ± 17.700	17.463 ± 8.853
F		1.451	0.020	1.626
*P*		0.237	0.980	0.199
HIV Viral Load(copies/ml)				
<20	50 (25.00)	4.720 ± 2.900	58.800 ± 13.438	18.840 ± 7.810
≥ 20	4 (2.00)	4.250 ± 2.630	57.250 ± 17.193	13.000 ± 6.976
Don't know	146 (73.00)	6.575 ± 4.347	54.164 ± 17.787	18.295 ± 10.351
F		4.398	1.443	0.669
*P*		0.014	0.239	0.513
Whether to get tested for HIV				
Yes	50 (25.00)	7.140 ± 4.233	51.340 ± 17.927	20.260 ± 10.462
No	150 (75.00)	5.707 ± 3.979	56.733 ± 16.299	17.680 ± 9.407
T		2.171	-1.976	1.632
*P*		0.031	0.050	0.104
Sexual Behavior				
Yes	80 (40.00)	5.713 ± 3.908	57.838 ± 16.762	17.588 ± 9.872
No	120 (60.00)	6.300 ± 4.192	53.750 ± 16.759	18.817 ± 9.626
T		-0.997	1.690	-0.876
*P*		0.320	0.093	0.382
Chronic Disease Conditions				
Yes	34 (17.00)	7.353 ± 4.625	56.412 ± 16.847	20.235 ± 12.196
No	166 (83.00)	5.801 ± 3.924	55.175 ± 16.880	17.934 ± 9.125
T		2.036	0.389	1.042
*P*		0.043	0.697	0.303
PSQI Score				
≤ 7	135 (67.50)	3.711 ± 1.816	57.785 ± 16.330	15.281 ± 7.670
>7	65 (32.50)	10.954 ± 2.987	50.400 ± 16.909	24.646 ± 10.508
T		-18.014	2.961	-6.410
*P*		<0.001	0.003	<0.001
MOS-SSS Score				
≤ 56	97 (48.50)	6.773 ± 4.097	41.557 ± 10.624	21.794 ± 9.773
>56	103 (51.50)	5.398 ± 3.971	68.408 ± 9.687	15.058 ± 8.495
T		2.410	-18.694	5.210
*P*		0.017	<0.001	<0.001
CES-D Score				
<16	91 (45.50)	4.088 ± 2.711	60.901 ± 17.187	10.000 ± 3.059
≥ 16	109 (54.50)	7.716 ± 4.304	50.780 ± 15.141	25.275 ± 7.688
T		-7.246	4.426	-19.019
*P*		<0.001	<0.001	<0.001

### Multiple linear regression analysis of depression symptoms in HIV/AIDS patients

The results of multiple linear regression analysis showed that among the relevant factors affecting the depressive symptoms of HIV/AIDS patients, the four variables, namely, thinness, obesity, sleep quality, and medical-social support, entered the regression model, and the results suggested that thinness (β = 0.161, *P* = 0.008), obesity (β = 0.186, *P* = 0.002), and sleep quality scores > 7 (Beta = 0.331, *P* < 0.001) all elevated depression scores and predisposed HIV/AIDS patients to depression symptoms. A medical social support score > 56 (β = -0.247, *P* < 0.001) decreased depression scores in HIV/AIDS patients and made them less prone to depression symptoms. These four variables explained 35.9% of the variance in depression scores. As for the correlation of antiretroviral treatment status on depression symptoms, the difference was not statistically significant (*P* > 0.05). From the standardized regression coefficients, the importance of the effects of the four variants ondepressive status was, in descending order, obesity, sleep quality score > 7, medical-social support score > 56, and thinness (Tables 2 and 3).

**Table 2:** Multiple linear regression dependent variable assignment

Variables	Assign a value
BMI	Normal weight = 0, Lean = 1, Overweight = 2, Obesity = 3
Health Status	Poor = 0, General = 1, Well = 2
Antiviral Treatment Status	Not treated = 0, Received treatment = 1
PSQI Score	≤ 7 = 0, >7 = 1
MOS-SSS Score	≤ 56 = 0, >56 = 1
CES-D Score	Raw value input

**Table 3:** Results of multiple linear regression analysis of depression symptoms in HIV/AIDS patients

Variability	B	SE	β	*t*	*P*	95%CI		VIF
						lower limit	limit	
Constant	23.537	3.430		6.863	<0.001	16.772	30.302	
BMI(VS: Normal)								
Lean	4.428	1.645	0.161	2.692	0.008	1.184	7.672	1.064
Overweight	0.616	1.532	0.024	0.402	0.688	-2.406	3.637	1.074
Obesity	9.218	2.940	0.186	3.135	0.002	3.418	15.017	1.053
Health Status(VS: Poor)								
General	-0.899	2.500	-0.044	-0.360	0.720	-5.831	4.033	4.541
Well	-3.685	2.507	-0.188	-1.470	0.143	-8.630	1.260	4.857
Antiviral Treatment Status (VS: No)								
Yes	-2.036	1.430	-0.086	-1.423	0.156	-4.857	0.786	1.095
PSQI Score (VS: ≤7)								
>7	6.852	1.316	0.331	5.208	<0.001	4.257	9.448	1.205
MOS-SSS Score (VS: ≤56)								
>56	-4.792	1.158	-0.247	-4.140	<0.001	-7.075	-2.509	1.062

Note: R^2^ = 0.359, adjusted R^2^ = 0.333, F = 13.393, *p* < 0.001, D-W value = 1.795, VIF < 5, no significant covariance in the independent variables, and residuals are normally distributed

### Correlation analysis of medical-social support, sleep quality and depression symptoms in HIV/AIDS patients

Pearson correlation analysis was used to measure the correlation between the variables. The correlation matrix of the variables is shown in Table 4, which showed that there was a two-by-two correlation between sleep quality, medical social support and depression symptoms in HIV/AIDS patients, with negative correlation between sleep quality and medical social support, medical social support and depression symptoms in HIV/AIDS patients (*P* < 0.05) and positive correlation between sleep quality and depression symptoms (*P* < 0.05).

**Table 4:** Correlation analysis of sleep quality, medical-social relations and depression symptoms in HIV/AIDS patients

Variability	Relevance(r)		
	medical-social support	sleep quality	depression symptoms
medical-social relation	1.000	-	-
sleep quality	-0.206*	1.000	-
depression symptoms	-0.357*	0.570*	1.000

Note: *, *p* < 0.05

### The mediating role of sleep quality

**Fig. 1:**
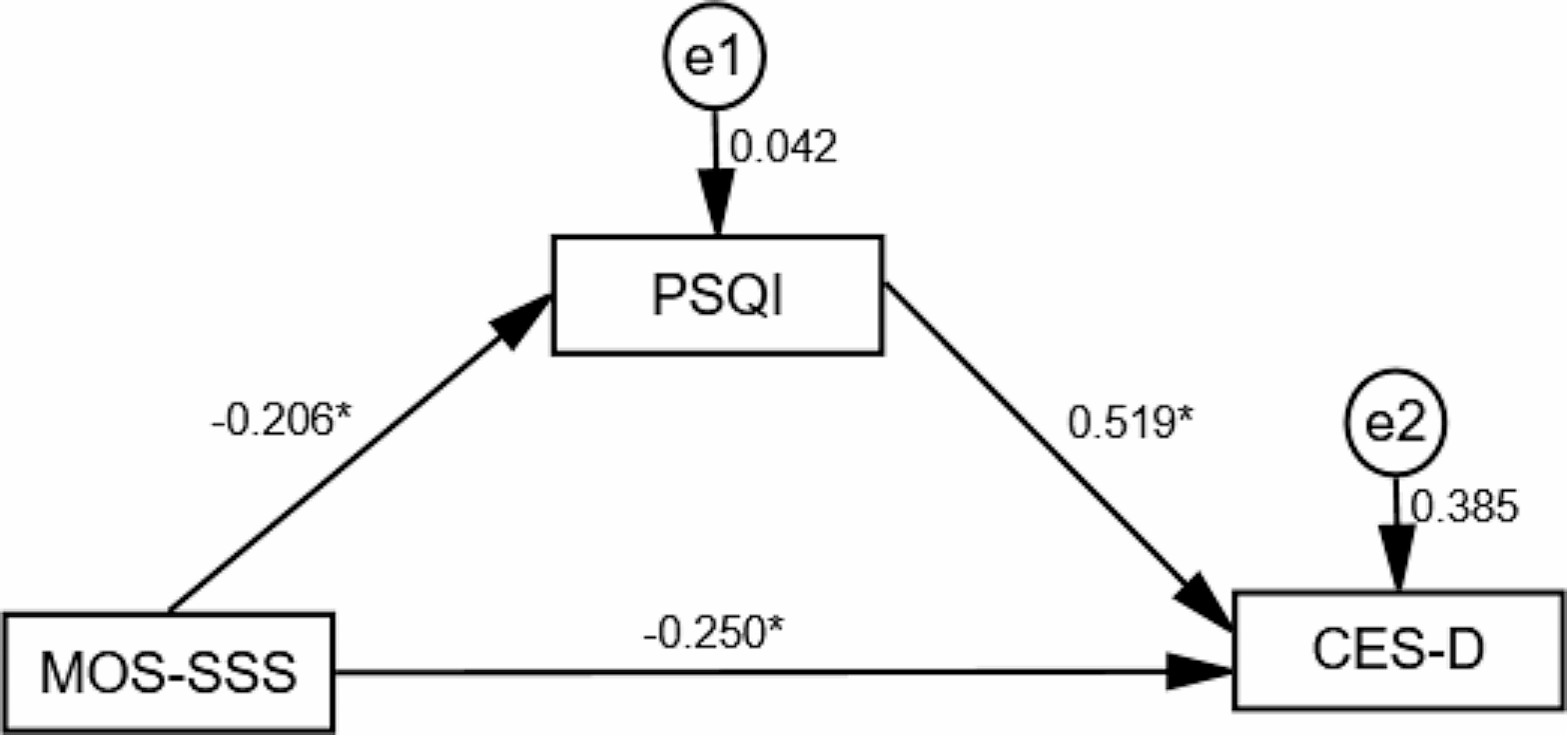
Schematic representation of the mediating effects of sleep quality

The results showed that medical-social support was negatively correlated with the effect of depression symptoms(β = -0.357, T = -5.378, *P* < 0.05), and when the mediating variable was added, medical-social support remained negatively correlated with depression symptoms (β = -0.250, T = -4.380, *P* < 0.05). Medical-social support was negatively correlated with sleep quality (β = -0.206, T = -2.963, *P* < 0.05), and sleep quality had a positive effect on depression symptoms (β = 0.519, T = 9.084, *P* < 0.05). (Table 5; Fig. 1)

**Table 5:** Mediation model test for sleep quality

	depression symptoms		depression symptoms		sleep quality	
	β	T	β	T	β	T
medical-social support	-0.250	-4.380*	-0.357	-5.378*	-0.206	-2.963*
sleep quality	0.519	9.084*	\	\	\	\
R^2^	0.385		0.127		0.042	
Adjusted R^2^	0.379		0.123		0.038	
F	61.675*		28.918*		8.781*	

Note: *, *p* < 0.05

Using Bootstrap resampling (5000 samples), the results showed a statistically significant difference in the direct effect of medical social support on depression symptoms as well as the indirect effect of depression symptoms(*P* < 0.05), suggesting that medical social support may affect depression not only directly, but also indirectly through the mediating effect of sleep quality. Adjusting the model by including BMI, health status and antiviral treatment status as covariates similarly showed the same results as above. Before adjustment, the direct and indirect mediating effects accounted for 69.90% and 30.10% of the total effect, respectively. After adjustment for the inclusion of covariates, the direct and indirect mediating effects accounted for 77.25% and 22.75% of the total effect, respectively (Table 6).

**Table 6:** Analysis of the mediating role of sleep quality in the relationship between medical social support and depressive symptoms

	Effect	se	T	*P*	LLCI	ULCI	relative effect value
Model 1^a^							
Total effect	-0.206	0.038	-5.378	<0.001	-0.282	-0.131	\
Direct effect	-0.144	0.033	-4.380	<0.001	-0.209	-0.079	69.90%
Indirect effect	-0.062	0.022	\	\	-0.106	-0.022	30.10%
Model 2^b^							
Total effect	-0.189	0.036	-5.207	<0.001	-0.261	-0.118	\
Direct effect	-0.146	0.033	-4.485	<0.001	-0.210	-0.082	77.25%
Indirect effect	-0.043	0.017	\	\	-0.078	-0.013	22.75%

*Note*: a, no adjustment; b, adjusted for inclusion of BMI, health status, and antiviral treatment as covariates

Table 6 Analysis of the mediating role of sleep quality in the relationship between medical social support and depressive symptoms Effect se T P LLCI ULCI relative effect value Model 1a Total effect -0.206 0.038 -5.378 ＜0.001 -0.282 -0.131 \Direct effect -0.144 0.033 -4.380 ＜0.001 -0.209 -0.079 69.90% Indirect effect -0.062 0.022 \\ -0.106 -0.022 30.10% Model 2b Total effect -0.189 0.036 -5.207 ＜0.001 -0.261 -0.118 \Direct effect -0.146 0.033 -4.485 ＜0.001 -0.210 -0.082 77.25% Indirect effect -0.043 0.017 \\ -0.078 -0.013 22.75% Note: a, no adjustment; b, adjusted for inclusion of BMI, health status, and antiviral treatment as covariates.

## Discussion

As the most common mental health challenge associated with HIV/AIDS patients, depression may even aggravate the disease [24]. In our study population, the mean depression score of HIV/AIDS patients was 18.33 ± 9.72, and the prevalence of depression sympyoms was 54.5%, which was higher than the global prevalence (31%) [25], and what was reported by Kim Madundo et al. (41%) [26] and Wang (47.7%) [27]. Possible reasons for this discrepancy may be related to timing, sample size, and differences in response to HIV infection around China, which influenced our subjects socially.

It has been shown that both leanness and obesity might have a negative impact on development of depression symptoms in HIV/AIDS patients. It’s widely acceptable that BMI is an important indicator of body nutritional status and a simple and cost-effective tool for monitoring the clinical response of HIV patients after initiation of ART [28]. Martinez [29] showed that higher BMI and adiposity slowed HIV disease progression and higher baseline BMI was associated with a lower risk of developing AIDS. In line with these previous studies mentioned above, our study also demonstrated that both malnutrition and obesity may contribute to the vulnerability of HIV/AIDS patients to depression symptoms [30, 31]. In contrast to Luo et al. [32], who found that obesity reduced the prevalence of depression in middle-aged and older men, Crisp [33 ] found a positive correlation between severe obesity and low levels of depression in men in their study. On the other hand, no association between obesity and depression or obesity increasing risk of depression were also reported [34, 35]. Therefore, more research is required to unvail the mystery of the relationship between obesity and depression. Low body weight and low BMI associated with disease wasting [36] may result in not only impaired immune recovery and increased mortality in the early stages of ART [37], but also the prevalence of menstrual disorders in PLWHs [38]. As a matter of fact, women are more likely to be depressive than men due to their physical hormonal fluctuations, such as hypersensitivity and hormonal changes during menopause [39]. In conclusion, there may be a strong association between obesity status and depression symptoms. BMI, as a tool to explore the relationship between obesity and depression symptoms, easily to be detected, may alert healthcare providers to the alteration in subject’s mood status at early stage.

In the present study, high medical-social support alleviated depression, similar to the study by Mohamud et al [40], where those with poor medical-social support were more likely to develop depression than those with moderate medical-social support. Though negative social behaviors may result in depression, it's still more likely that people with HIV try to avoid seeking help from others due to social stigma, which inevidently increases their loneliness, isolation, and depression. Data suggests that 41.7% of the people with HIV have experienced HIV-related discrimination, and more than 76% report that their family members are even been discriminated against [41]. Moreover, increased emotional, financial, and physical burden of stigmatization were observed [42]. Despite legal access to health care, employment and education, discrimination still persists in both medical and non-medical resources [12, 42]. Stigmatization, fatigue feelings, worthlessness, shame, and fear of HIV exposure, isolation, and hopelessness may further exacerbate depression which in turn may lead to ultimate treatment failure. In China, where most activities are culturally family-centered, it's much easier for most patients to receive or solicit support from their families. Hence, a supportive and active family environment may positively affect the mood of patients, prevent social isolation, and promote healthy sleep habits [11]. Good social support may enable PLWH to better cope with the negative effects of stress and modulate themselves to physical discomfort [43]. To summarize, adequate and ready medical-social support may play a critical role in assisting the patients in building up their confidence to live optimistically, maintaining a good psychological state, and ultimately improving treatment outcomes.

PLWH frequently claimed sleep disturbances. In our study, sleep quality was not only a positive predictor of depressive symptoms, but also had a mediating effect in medical social support and depressive symptoms. This suggests that medical social support can both directly and indirectly influence depressive symptoms in HIV/AIDS patients by affecting sleep quality. In China, where AIDS is still a highly stigmatized disease, the impact of HIV infection on psychological status, as well as the prejudice and social stigma followed, may place PLWH at a higher risk of depression which may subsequently induce sleep disorders [44]. Sleep disorders may, in turn, lead to aggravation of depressive symptoms in PLWH, which was consistent with that participants suffering from depression were 4.44 times more likely to experience poor sleep quality compared to those without depression [11]. The possible reason for this is that PLWH are more psychologically stressed after HIV infection and mostly reluctant to resort to others, which lead to depression and other adverse emotions and poor sleep quality. Whereas high medical social support may alleviate depressive symptoms by increasing positive emotions in HIV/AIDS patients, thereby improving sleep quality, it may provide a new way of thinking about alleviating depressive symptoms in HIV/AIDS patients in Guilin. Thus, addressing sleep disorders may alleviate mental illness, and treating depressive symptoms may improve sleep quality, while increasing the level of medical-social support in PLWH is needed.

Our study described the current status of depression among people living with HIV/AIDS (PLWH) in Guilin and the mediating role of sleep quality in medical-social support and depressive symptoms. And the results suggested that there were two ways to reduce the depressive symptoms of PLWH: (1) Taking measures to improve the sleep quality of PLWH, such as promoting a more regular lifestyle. (2) Improving medical-social support for PLWH, such as increasing more daily social contacts. PLWHs often have difficulty in obtaining social support directly from friends and relatives, which highlights the psychological support and care provided by health care providers for PLWHs. It is very feasible for health service staff, especially those for PLWH, to utilize their professional knowledge and exclusive channels to provide adequate and sustainable services.

Limitation.

Although our study provides some valuable insights into the beneficial effects of medical-social support on depression symptoms, there are still some limitations worth being taken into account when the current results were interpreted. First, because this was a cross-sectional study, it was not possible to determine causality. Second, the reliance on self-reported data for certain variables may introduce potential recall bias, which may affect the accuracy and completeness of the data collected. Third, the application of convenience sampling methods may also lead to sample selection bias, limiting the generalizability of our findings on other populations. Fourth, depressive symptoms are conceptualized differently from depression, and research on depressive symptoms may not fully guide interventions for depression. Therefore, in future studies, more sound research design and sampling methods to increase the sample size of the study are required to obtain more reliable and generalizable results. Secondly, clinical diagnosis with the aim that the results can better guide interventions for depression may be considered to introduce. Finally, the results of this study need to be further confirmed by prospective follow-up studies with large samples.

## Conclusion

In summary, the prevalence of depression symptoms is high among HIV/AIDS patients in Guilin City. The depressive symptoms of PLWHs may be related to sleep quality and medical-social support. In addition, sleep quality may partially mediate the relationship between medical-social support and depressive symptoms. Therefore, interventions to improve sleep quality and medical-social support may have the potential to reduce severity of the depressive symptomsof HIV/AIDS patients. 

### Electronic Supplementary Material

Below is the link to the electronic supplementary material.

Supplementary Material 1

## Data Availability

The datasets used and/or analysed during the current study are available from the corresponding author upon reasonable request.
