# Supplementary material for: Sleep quality mediates the effect of medical social support on depression symptoms in patients with HIV/AIDS
Source: BMC Public Health. 2024 May 28;24:1429. doi: 10.1186/s12889-024-18174-w (PMC11134677; doi:10.1186/s12889-024-18174-w)
Supplement: Supplementary file 1 — Supplementary Material 1 [file 12889_2024_18174_MOESM1_ESM.pdf]

Table 1 Comparison of general conditions of HIV/AIDS patients

Characteristics	N (%)	Sleep quality	Medical-social support	Depression symptoms
Genders				
Male	129 (64.50)	5.705 ± 4.223	54.977 ± 17.512	17.519 ± 9.547
Female	71 (35.50)	6.718 ± 3.750	56.127 ± 15.634	19.789 ± 9.925
T		-1.687	-0.461	-1.586
P		0.093	0.645	0.114
Age, (yeas)				
＜50	85 (42.50)	5.612 ± 3.964	55.576 ± 17.673	18.776 ± 8.970
≥50	115 (57.50)	6.400 ± 4.150	55.243 ± 16.271	17.991 ± 10.264
T		-1.353	0.138	0.564
P		0.178	0.890	0.574
Ethnicity				
Han	152 (76.00)	6.237 ± 4.186	55.118 ± 17.687	17.816 ± 10.029
Yao	39 (19.50)	5.462 ± 3.409	55.615 ± 14.100	20.154 ± 8.509
Others	9 (4.50)	5.778 ± 5.094	58.889 ± 13.724	19.000 ± 9.247
F		0.581	0.216	0.920
P		0.560	0.806	0.400
Marital status				
With partner	118 (59.00)	5.415 ± 3.440	57.602 ± 16.226	18.000 ± 9.806
No partner	82 (41.00)	7.000 ± 4.725	52.195 ± 17.287	18.793 ± 9.634
T		-2.597	2.256	-0.566
P		0.010	0.025	0.572
Education				
Elementary school and below	79 (39.50)	7.241 ± 4.282	52.152 ± 17.425	19.658 ± 10.646
Junior High School	83 (41.50)	5.422 ± 3.646	55.795 ± 16.164	18.241 ± 9.636
High School and above	38 (19.00)	5.026 ± 4.057	61.211 ± 15.755	15.737 ± 7.258
F		5.807	3.863	2.117
P		0.004	0.023	0.123
Career				
Farmers	148 (74.00)	5.939 ± 3.767	55.041 ± 16.537	17.905 ± 9.444
Workers/services	25 (12.50)	6.840 ± 5.528	58.240 ± 19.361	19.280 ± 10.722
Others	27 (13.50)	6.037 ± 4.283	54.630 ± 16.397	19.741 ± 10.424
F		0.519	0.415	0.543
P		0.596	0.661	0.582
Health Status				
Poor	13 (6.50)	10.385 ± 4.292	50.077 ± 19.902	26.846 ± 10.439
General	71 (35.50)	7.268 ± 4.323	53.352 ± 15.123	20.761 ± 10.687

Well	116 (58.00)	4.845 ± 3.320	57.224 ± 17.348	15.879 ± 8.041
F		18.246	1.872	12.116
P		＜0.001	0.157	＜0.001
Income (CNY)				
＜1000	64 (32.00)	6.328 ± 4.346	52.266 ± 17.862	17.422 ± 11.420
1001~2000	59 (29.50)	5.763 ± 3.612	55.288 ± 16.565	18.356 ± 8.570
2001~3000	48 (24.00)	5.396 ± 3.902	57.771 ± 14.206	19.229 ± 9.210
＞3000	29 (14.50)	7.207 ± 4.562	58.517 ± 18.652	18.759 ± 8.943
F		1.392	1.397	0.339
P		0.247	0.245	0.797
Household Registration				
Rural	161 (80.50)	6.019 ± 3.957	54.373 ± 17.107	18.453 ± 10.008
City	39 (19.50)	6.256 ± 4.610	59.564 ± 15.176	17.795 ± 8.520
T		-0.326	-1.736	0.379
P		0.745	0.084	0.705
BMI (kg/m^2)				
Lean	29 (14.50)	5.966 ± 3.986	54.276 ± 16.349	21.690 ± 10.794
Normal weight	128 (64.00)	6.188 ± 4.091	55.063 ± 17.548	17.406 ± 9.367
Overweight	35 (17.50)	5.171 ± 3.258	58.029 ± 14.199	16.571 ± 8.158
Obesity	8 (4.00)	8.375 ± 6.653	53.000 ± 19.413	28.500 ± 10.406
F		1.468	0.395	5.145
P		0.224	0.757	0.002
Route of Infection				
Heterosexual transmission	177 (88.50)	6.051 ± 4.048	55.542 ± 16.627	17.989 ± 9.805
Homosexual transmission	18 (9.00)	6.278 ± 4.638	58.667 ± 17.918	21.167 ± 9.044
Non-sexual transmission	5 (2.50)	5.800 ± 3.962	38.000 ± 12.247	20.000 ± 8.746
F		0.036	3.077	0.949
P		0.965	0.048	0.389
Antiviral Treatment Status				
Not treated	43 (21.50)	7.744 ± 4.909	55.930 ± 17.856	21.674 ± 10.884
Received treatment	157 (78.50)	5.605 ± 3.710	55.236 ± 16.606	17.408 ± 9.201
T		2.657	0.239	2.587
P		0.010	0.811	0.010
Duration of Infection (years)				
＜1	42 (21.00)	7.762 ± 5.169	58.619 ± 18.285	21.071 ± 11.788
1~2	12 (6.00)	5.917 ± 4.641	57.667 ± 13.013	18.500 ± 13.070
3~5	42 (21.00)	5.881 ± 3.717	54.619 ± 17.518	17.595 ± 8.402
＞5	104 (52.00)	5.471 ± 3.492	54.125 ± 16.363	17.490 ± 8.767

F		3.299	0.811	1.464
P		0.021	0.489	0.226
Most Recent CD4+ Count(cells/mm3)				
＜200	47 (23.50)	6.915 ± 4.496	55.000 ± 16.926	20.532 ± 11.521
200-409	71 (35.50)	5.972 ± 3.633	55.634 ± 15.972	17.859 ± 9.288
410-1590	82 (41.00)	5.659 ± 4.176	55.390 ± 17.700	17.463 ± 8.853
F		1.451	0.020	1.626
P		0.237	0.980	0.199
HIV Viral Load（copies/ml）				
＜20	50 (25.00)	4.720 ± 2.900	58.800 ± 13.438	18.840 ± 7.810
≥20	4 (2.00)	4.250 ± 2.630	57.250 ± 17.193	13.000 ± 6.976
Don't know	146 (73.00)	6.575 ± 4.347	54.164 ± 17.787	18.295 ± 10.351
F		4.398	1.443	0.669
P		0.014	0.239	0.513
Whether to get tested for HIV				
Yes	50 (25.00)	7.140 ± 4.233	51.340 ± 17.927	20.260 ± 10.462
No	150 (75.00)	5.707 ± 3.979	56.733 ± 16.299	17.680 ± 9.407
T		2.171	-1.976	1.632
P		0.031	0.050	0.104
Sexual Behavior				
Yes	80 (40.00)	5.713 ± 3.908	57.838 ± 16.762	17.588 ± 9.872
No	120 (60.00)	6.300 ± 4.192	53.750 ± 16.759	18.817 ± 9.626
T		-0.997	1.690	-0.876
P		0.320	0.093	0.382
Chronic Disease Conditions				
Yes	34 (17.00)	7.353 ± 4.625	56.412 ± 16.847	20.235 ± 12.196
No	166 (83.00)	5.801 ± 3.924	55.175 ± 16.880	17.934 ± 9.125
T		2.036	0.389	1.042
P		0.043	0.697	0.303
PSQI Score				
≤7	135 (67.50)	3.711 ± 1.816	57.785 ± 16.330	15.281 ± 7.670
＞7	65 (32.50)	10.954 ± 2.987	50.400 ± 16.909	24.646 ± 10.508
T		-18.014	2.961	-6.410
P		＜0.001	0.003	＜0.001
MOS-SSS Score				
≤56	97 (48.50)	6.773 ± 4.097	41.557 ± 10.624	21.794 ± 9.773
＞56	103 (51.50)	5.398 ± 3.971	68.408 ± 9.687	15.058 ± 8.495
T		2.410	-18.694	5.210

P		0.017	＜0.001	＜0.001
CES-D Score				
＜16	91 (45.50)	4.088 ± 2.711	60.901 ± 17.187	10.000 ± 3.059
≥16	109 (54.50)	7.716 ± 4.304	50.780 ± 15.141	25.275 ± 7.688
T		-7.246	4.426	-19.019
P		＜0.001	＜0.001	＜0.001

Table 6 Analysis of the mediating role of sleep quality in the relationship between medical social support and depressive symptoms

	Effect	se	T	*P*	LLCI	ULCI	relative effect value
Model 1[a]							
Total effect	-0.206	0.038	-5.378	＜0.001	-0.282	-0.131	\
Direct effect	-0.144	0.033	-4.380	＜0.001	-0.209	-0.079	69.90%
Indirect effect	-0.062	0.022	\	\	-0.106	-0.022	30.10%
Model 2[b]							
Total effect	-0.189	0.036	-5.207	＜0.001	-0.261	-0.118	\
Direct effect	-0.146	0.033	-4.485	＜0.001	-0.210	-0.082	77.25%
Indirect effect	-0.043	0.017	\	\	-0.078	-0.013	22.75%

Note: a, no adjustment; b, adjusted for inclusion of BMI, health status, and antiviral treatment as covariates.

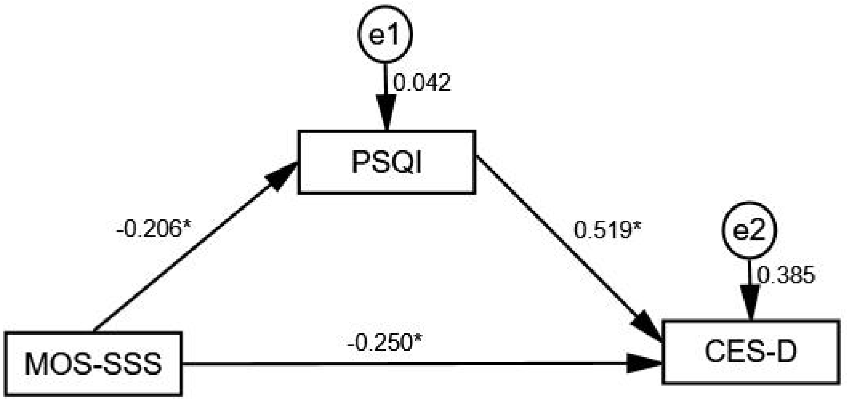

Figure 1 Schematic representation of the mediating effects of sleep quality
